# Mortality in Paris

**Published:** 1871-02

**Authors:** 


MORTALITY IN PARIS.—A besieged city always affords one of the most striking exhibitions of the horrors of war. The death-rate in Paris during the month of November was four hundred a week in excess of the average, and it must have increased rather than diminished since that time. In the second week of November, the mortality of small-pox, which declined in August, had risen to four hundred and nineteen.—*Ibid.*

